# Stent fracture and embolization: an unusual late complication of neonatal coarctation of aorta stenting with bioresorbable vascular scaffold

**DOI:** 10.1186/s43044-026-00778-9

**Published:** 2026-09-14

**Authors:** Qayoom Yousuf, Aamir Rashid, Sameer Purra, Syed Bilal, Mehraj Khan

**Affiliations:** Sher-i-Kashmir Institute of Medical Sciences, Srinagar, India

**Keywords:** Coarctation of aorta, Neonatal stenting, Bioresorbable vascular scaffold, Stent fracture

## Abstract

**Background:**

Critical neonatal coarctation of the aorta (CoA) may present with cardiogenic shock and severe ventricular dysfunction, rendering immediate surgical repair high risk. In such situations, transcatheter interventions may be used as a temporary stabilizing strategy.

**Case presentation:**

A 10-day-old male neonate with critical CoA presented in cardiogenic shock and severe left ventricular dysfunction. Balloon angioplasty resulted in only transient improvement due to elastic recoil, necessitating rescue implantation of a bioresorbable vascular scaffold. This led to rapid haemodynamic stabilization and recovery of ventricular function. Follow-up computed tomography angiography revealed late scaffold fracture with distal embolization to the proximal abdominal aorta. The child remained clinically stable and subsequently underwent successful elective surgical repair.

**Conclusion:**

Although bioresorbable vascular scaffolds can provide short-term haemodynamic stabilization in critically ill neonates with CoA, their use is limited by mechanical vulnerability in the neonatal aorta, as illustrated by late scaffold fracture in this case. Their role should therefore be highly selective, with close imaging surveillance and early definitive surgical correction.

## Background

Coarctation of the aorta (CoA) accounts for approximately 6–8% of all congenital heart defects. While some patients may remain asymptomatic until later childhood or adulthood, severe neonatal CoA can present with congestive heart failure, cardiogenic shock, or multi-organ dysfunction; requiring urgent intervention. Surgical repair remains the standard of care, offering definitive correction [1]. However in specific high-risk settings - such as profound ventricular dysfunction, extreme prematurity, or severe comorbidities, primary surgery may be associated with prohibitive risk and stent implantation can provide immediate hemodynamic stabilization, unload the left ventricle to permit myocardial recovery, somatic growth and serve as a bridge to safer elective repair [2–5]. Due to the limited availability of dedicated neonatal CoA stents, off-label use of coronary and peripheral stents is commonly employed for this purpose [6, 7]. Experience with bioresorbable vascular scaffolds (BVS) in neonatal coarctation is limited to isolated case reports, in which they have been used as a bailout option to achieve temporary luminal support [8, 9]. Although restenosis and reintervention are anticipated limitations of this approach, stent fracture and embolization are infrequently reported complications [10]. We report an unusual case of stent fracture with distal embolization following implantation of a BVS for critical neonatal CoA as a bailout intervention.

## Case presentation

A 10-day-old male neonate, delivered by cesarean section, presented with poor feeding, tachypnea, and progressive lethargy worsening over 24 h. On admission, he was in cardiogenic shock with features of congestive heart failure and weighed 2.6 kg. Examination revealed feeble upper-limb pulses with absent femoral pulses. Transthoracic echocardiography showed severe left ventricular systolic dysfunction with ejection fraction (EF) of 20% and a discrete critical coarctation distal to the left subclavian artery, with a peak doppler gradient of 50 mmHg and diastolic tailing. Prostaglandin E1 and decongestive therapy were initiated, and balloon angioplasty was planned as a bridging strategy.

**Fig. 1:**
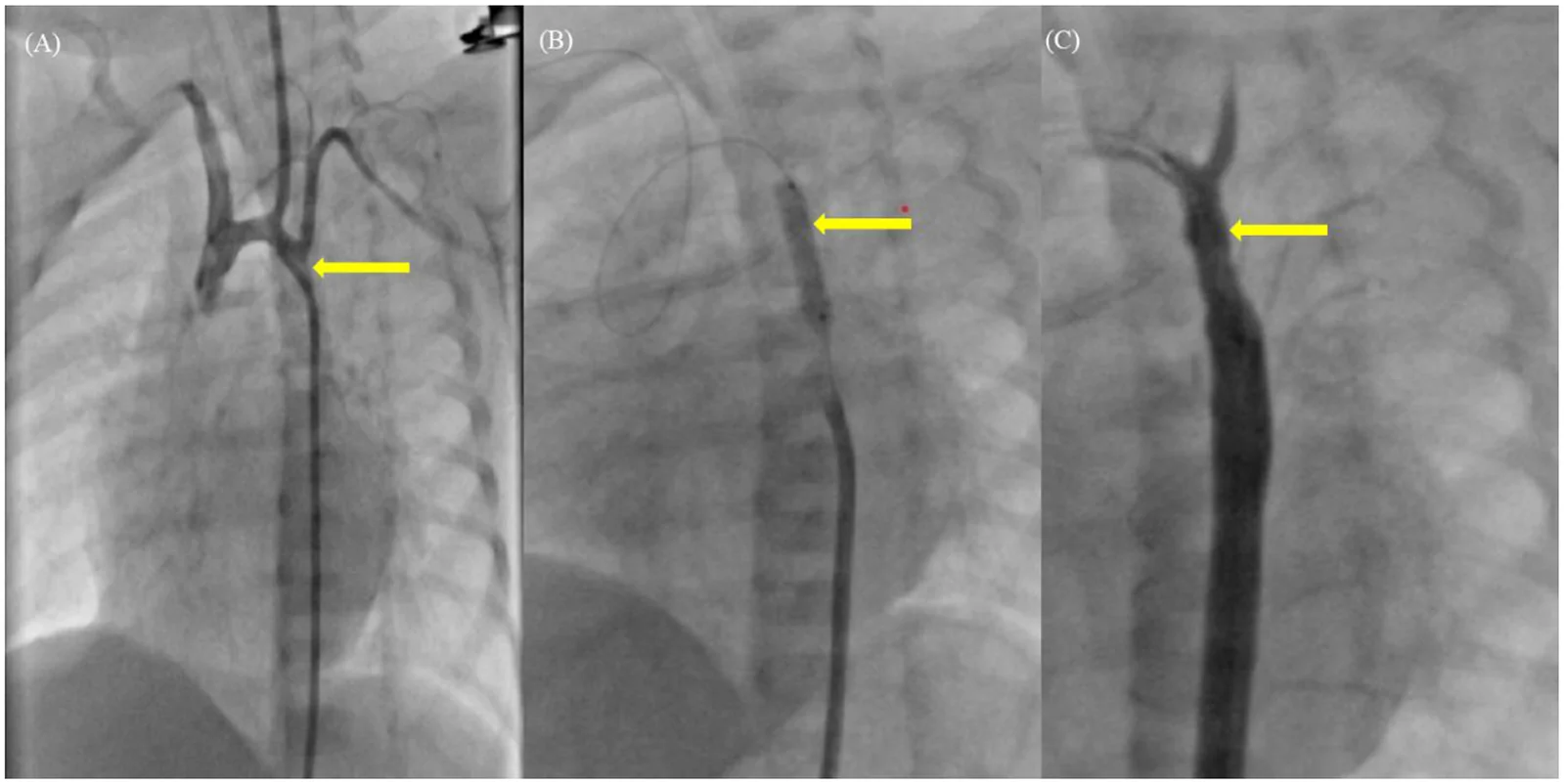
Angiography images (**A**) LAO 30 view showing severe coarctation of aorta (yellow arrow). (**B**) LAO 30 view showing balloon dilatation of the coarct segment (yellow arrow). (**C**) LAO 30 view showing good post balloon angioplasty result at coarct site (yellow arrow)

The procedure was performed under elective intubation via transfemoral arterial access using a 5 F sheath and 5 F JR guide, with a 4 F JR catheter for angiography (Fig. 1A). The aortic isthmus measured approximately 3.5 mm on echocardiography and angiography, with mild, hemodynamically insignificant transverse arch hypoplasia. Sequential balloon dilatation was performed using 3.0 × 12 mm and 3.5 × 12 mm non-compliant coronary balloons at 12 atmospheres (Fig. 1B and C), resulting in a reduction of the gradient to 17 mmHg and improvement in EF to 40%.

**Fig. 2:**
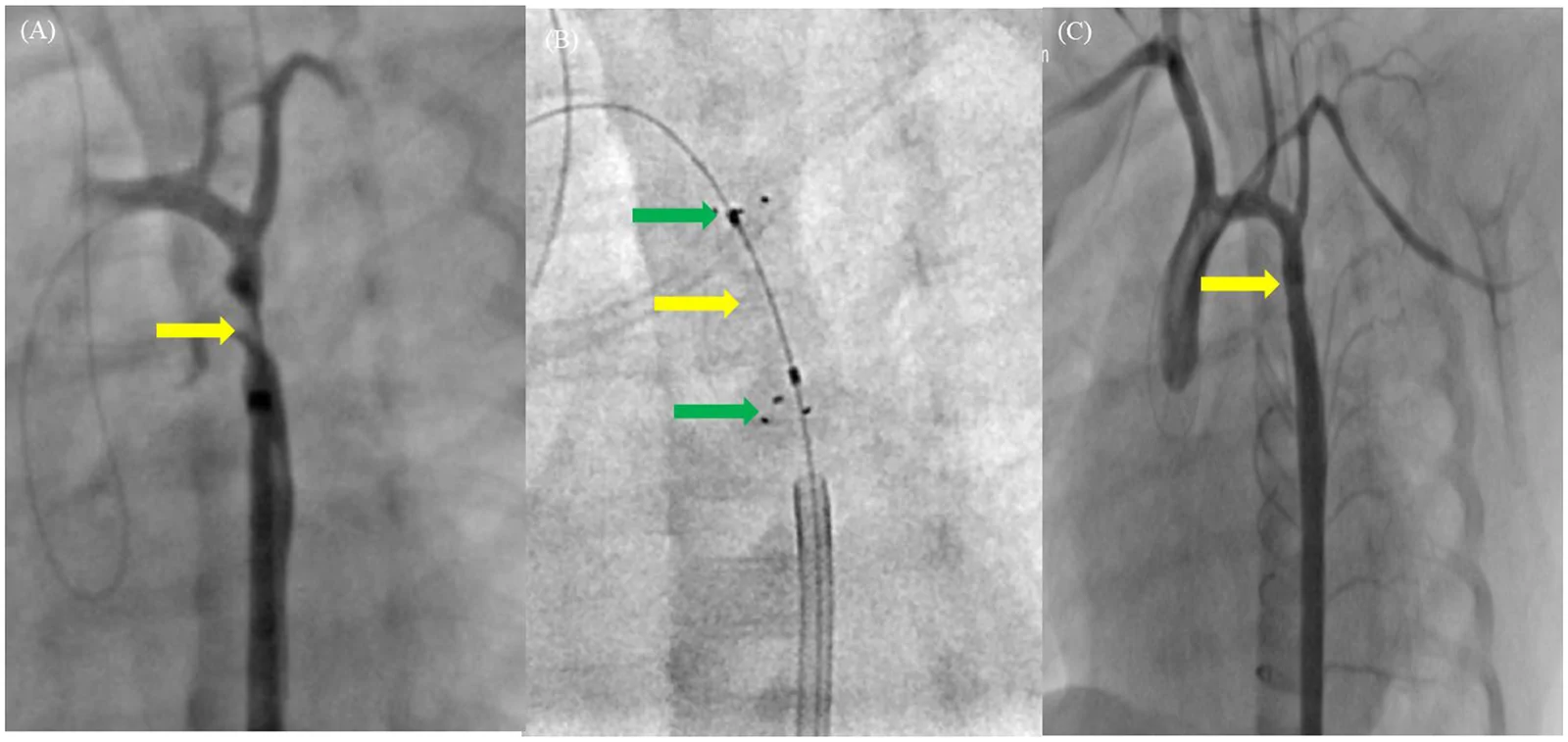
Angiography images (**A**) LAO 30 view showing Recoarctation due to elastic recoil (yellow arrow) (**B**) Bioresorbable vascular scaffold (MeRes 100) deployed across the coarct segment with visible proximal and distal stent markers (green arrows) and invisible stent body (yellow arrow). (**C**) LAO 30 view post- deployment showing the BVS well expanded across the coarctation segment with good luminal patency and antegrade flow (yellow arrow)

Serial echocardiography demonstrated transient improvement, and a trial of extubation was attempted approximately 30 min after angioplasty. During extubation, the infant developed acute pulmonary edema with echocardiographic evidence of elastic recoil, recurrent obstruction (peak gradient 42 mmHg), and worsening ventricular dysfunction (EF 25%), prompting urgent reintervention. Repeat angiography confirmed recoil (Fig. 2A), and a 3.5 × 15 mm MeRes100 (Meril Life Sciences, India) bioresorbable vascular scaffold (BVS) was implanted at 9 atmospheres (Fig. 2B), followed by post-dilatation with a 4.0 × 10 mm non-compliant balloon at 12 atmospheres. Final angiography demonstrated good luminal expansion with a residual gradient of 15 mmHg (Fig. 2C).

**Fig. 3:**
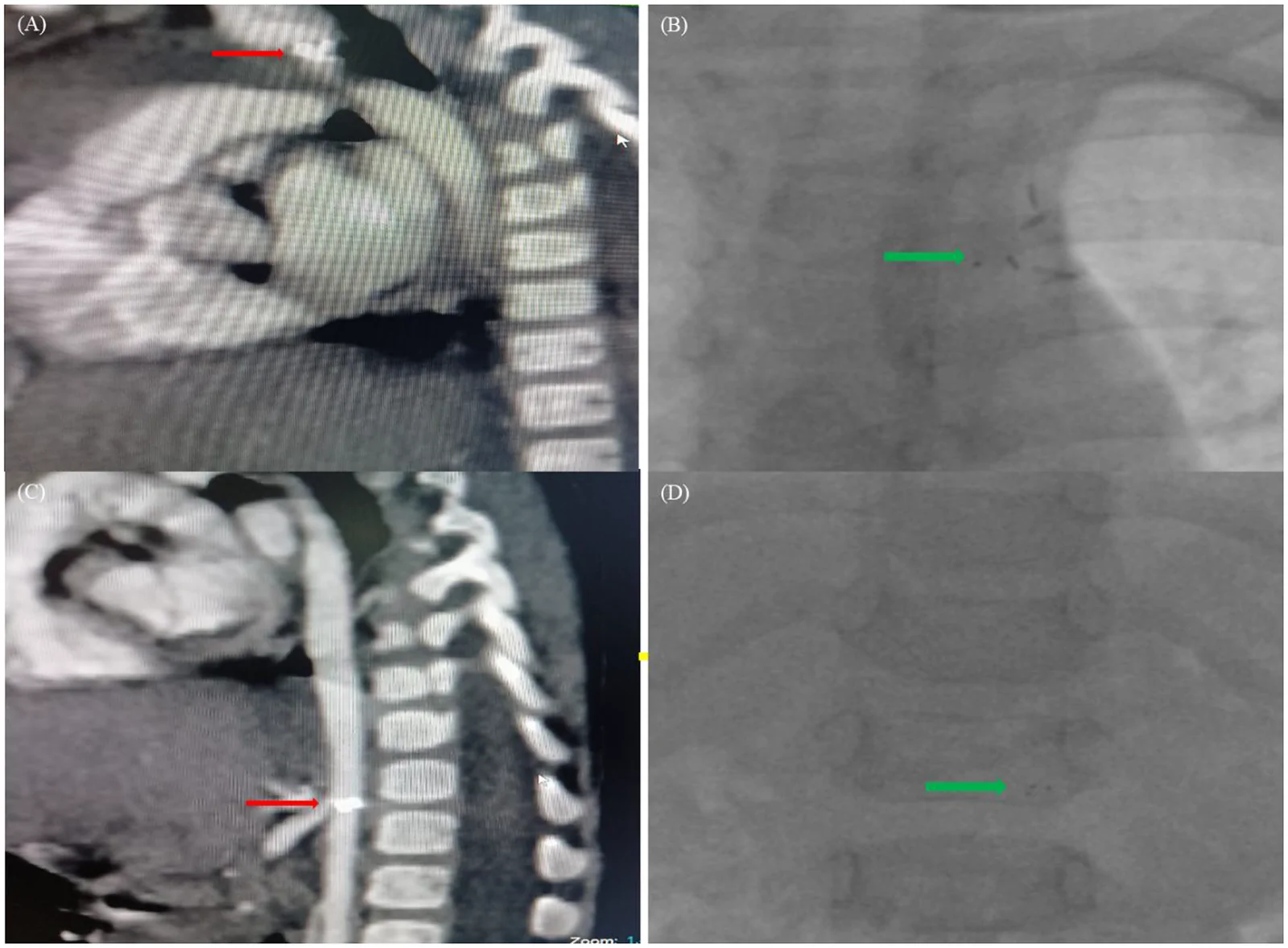
(**A**) CT angiogram, sagittal reconstruction, showing proximal BVS fragment near the isthmus (red arrow). (**B**) Fluoroscopic AP view showing proximal stent markers (green arrow) near the isthmus (fluro taken after surgery). (**C**) CT angiogram, sagittal reconstruction, showing distal BVS fragment in the proximal abdominal aorta (red arrow). (**D**) Fluoroscopic AP view showing distal stent markers in the proximal abdominal aorta

The patient stabilized rapidly, was extubated within 24 h, and later discharged on aspirin, diuretics and enalapril with improved LV function (EF 45%). Femoral artery patency was preserved. At follow-up, ventricular function normalized (EF 62%); however, at 6 months, echocardiography showed recurrent gradient of 55 mmHg. CT angiography revealed fracture of the scaffold with distal embolization to the proximal abdominal aorta, confirmed fluoroscopically (Fig. 3A and C). The child remained asymptomatic and subsequently underwent elective surgical repair at 8 months of age with an 8-mm Dacron interposition graft, with the embolized fragment left in situ anticipating bioresorption (Fig. 3B and D). At 2-year follow-up, he was normotensive, off medications, and had normal ventricular function with a residual gradient of 15 mmHg.

## Discussion

Stenting is a feasible option in selected high-risk infants with critical CoA when primary surgery carries prohibitive risk, particularly in the presence of severe ventricular dysfunction. Several reports support its use in infants younger than 3 months, including very low-birth-weight neonates, as an effective bridging strategy to definitive repair [2–7]. In this setting, stent implantation provides rapid relief of obstruction, restores systemic perfusion, and facilitates ventricular recovery and clinical stabilization. More recently, the Renata Minima stent has received U.S. Food and Drug Administration approval for use in selected pediatric patients with native or recurrent CoA, reflecting progress toward dedicated, redilatable stent platforms.

Comparative studies suggest that a staged stent–surgery approach yields outcomes comparable to primary neonatal surgery, with the additional benefit of improved myocardial remodeling before definitive repair [5]. However, neonatal stenting is associated with important limitations, including femoral artery injury, restenosis, aneurysm formation, and the frequent need for early surgical removal due to limited expandability in a growing aorta [4, 6, 7]. 

In the present case, a sirolimus-eluting, 100 μm thin-strut BVS (MeRes100) was used as a bailout measure after failed balloon angioplasty in the setting of severe ventricular dysfunction. The device was selected with the specific intention of providing luminal support through the period of haemodynamic vulnerability and then resorbing by the time of elective surgical repair and subsequent aortic growth, thereby avoiding a permanent implant. A coronary bare-metal or drug-eluting stent of deliverable calibre in a 2.6 kg neonate can be redilated to no more than 5–6 mm and would have become a fixed obstruction in a growing aorta, requiring explantation at definitive repair [6, 7]. Temporary scaffolding with bioabsorbable devices has previously been described in neonatal recoarctation with short-term feasibility [8], and polymer-based scaffolds have been reported in isolated cases of native CoA, although concerns regarding radial strength and mechanical durability in large elastic vessels have been highlighted [9]. 

In our patient, the scaffold provided immediate relief of obstruction, mitigated elastic recoil, and enabled ventricular recovery, thereby serving its intended role as a bridge to surgery. However, polymer-based BVS have inferior radial strength and limited resistance to fatigue in the high-pressure, pulsatile aortic environment, which likely contributed to scaffold fracture at 6 months in this case. Fracture represents a pathological mechanical failure, distinct from the expected gradual bioresorption of the scaffold.

Published experience with bioresorbable scaffolds in the neonatal aorta, although available, is limited to a small number of case reports with short follow-up, in which fracture and embolisation were not reported [8, 9]. Fragmentation with distal migration has, however, been described elsewhere in paediatric use, including embolisation of iron scaffold fragments to distal pulmonary branches following ductal stenting, without clinical sequelae [11], and loss of mechanical integrity within weeks has been observed across several paediatric applications [12]. The evidence base in this setting therefore remains considerably narrower than that for metallic stents.

Although distal embolization of a fractured segment was clinically silent in this child, it underscores the potential for serious complications, including distal ischemia or embolic events. This highlights the need for close surveillance with cross-sectional imaging in neonates undergoing off-label stenting, as clinical stability alone does not ensure long-term device integrity. Ultimately, elective surgical repair was successfully performed under more favourable conditions, reinforcing the role of BVS, when used, as a temporary and highly selective rescue strategy rather than a durable solution for neonatal CoA.

## Conclusion

BVS may provide temporary hemodynamic stabilization as a bailout measure in critically ill neonates with CoA when balloon angioplasty fails and immediate surgery carries prohibitive risk. However, current-generation polymer-based BVS have important mechanical limitations in the neonatal aorta, including reduced radial strength and vulnerability to fatigue-related fracture in a high-pressure, pulsatile environment. Their use must therefore be highly selective, individualized, and restricted to exceptional rescue situations. Close surveillance with imaging is essential, as clinical stability alone does not preclude late scaffold-related complications.

## Data Availability

No datasets were generated or analysed during the current study.
